# miRNAs signature as potential biomarkers for cervical precancerous lesions in human papillomavirus positive women

**DOI:** 10.1038/s41598-023-36421-9

**Published:** 2023-06-17

**Authors:** Martha I. González-Ramírez, Yurley T. Cardona, María C. Agudelo, Carolina López, Juan J. Florez-Acosta, Samuel Agudelo-Gamboa, Jone Garai, Li Li, Carlos A. Orozco-Castaño, Jovanny Zabaleta, Gloria I. Sánchez

**Affiliations:** 1 Infection and Cancer Group, School of Medicine, Universidad de Antioquia, Cra 51D No 62-29 Lab 219, Medellín, 050010 Antioquia Colombia; 2 Department of Pathology, Universidad de Antioquia, Medellín, 050010 Antioquia Colombia; 3 Stanley S. Scott Cancer Center, Louisiana State University Health Sciences Center, New Orleans, LA 70112 USA; 4 School of Health and Sport Sciences, Fundación Universitaria del Área Andina, Bogotá, 110111 Cundinamarca Colombia; 5 Department of Interdisciplinary Oncology, Louisiana State University Health Sciences Center, New Orleans, LA 70112 USA

**Keywords:** Cancer, Molecular biology, Biomarkers, Molecular medicine, Oncology

## Abstract

Biomarkers to identify women at risk of cervical cancer among those with high-risk HPV infection (hrHPV+) are needed. Deregulated expression of microRNAs (miRNAs) contributes to hrHPV-induced cervical carcinogenesis. We aimed at identifying miRNAs with the capacity to distinguish high (CIN2+) and low (≤ CIN1) grade cervical lesions. We sequenced miRNA libraries from Formalin-Fixed Paraffin-Embedded (FFPE) tissues from women with CIN2+ (n = 10) and age-matched women with ≤ CIN1 (n = 10), randomly and retrospectively selected from a trial that followed women for 24 months after a hrHPV+ test at the screening visit. Five miRNAs differentially expressed were validated by RT-qPCR in an independent set of FFPE tissues with a reviewed diagnosis of CIN2+ (n = 105) and ≤ CIN1 (n = 105). The Ingenuity Pathway Analysis (IPA) was conducted to identify mRNAs inversely correlated with the top 25 differentially expressed miRNAs. Inverse correlations with 401 unique mRNA targets were identified for fourteen of the top 25 differentially expressed miRNAs. Eleven of these miRNAs targeted 26 proteins of pathways deregulated by HPV E6 and E7 oncoproteins and two of them, miR-143-5p and miR-29a-3p, predicted CIN2+ and CIN3+ in the independent validation by RT-qPCR of FFPE tissues from hrHPV-positive women.

## Introduction

Cervical cancer is the fourth most frequently diagnosed cancer and the fourth leading cause of cancer death in women, with an estimated 604,000 new cases and 342,000 deaths worldwide in 2020. Around 90% of these cases and deaths occur in Asia, Africa, Latin America, and the Caribbean regions^1^. Although cervical cancer mortality rates have declined in most areas of the world for the past few decades, they remain disproportionately high in underserved populations mostly living in low-middle income countries (LMICs), but also in low resource settings of high-income countries (HICs) as the United States and Europe, where the cervical cancer death rate is twofold higher among women residing in high-poverty versus low-poverty areas^2,3^. High-risk Human Papillomavirus (hrHPV), a common sexually transmitted infection worldwide, is the cause of almost all cervical cancer. Nearly 90% of hrHPV infections are transient and only a small proportion of hrHPV-positive women develop persistent infection and cervical intraepithelial neoplasia (CIN) grade 2 or 3 (CIN2 and CIN3, respectively) which may if left untreated, progress to cancer^4^. However, identifying and treating properly the low proportion of hrHPV+ women at risk of high-grade disease (CIN2, CIN3) or cancer requires resource-consuming screening programs, which have not been possible to implement in low-resource settings. hrHPV DNA testing is the best alternative for screening because it has more than 95% both sensitivity and negative predictive value for detecting CIN2+ and CIN3+^5^, which, permits longer screening intervals and more cost-effective screening programs^6^. Even further, the logistics attributes and robustness of the HPV test offer the opportunity for self-sampling, a modality that improves the reach of women in low resource settings^7^. However, hrHPV testing has low specificity, increasing the number of women for follow-up and leading to overtreatment of women without risk of progressive disease^8^. If miRNAs are proven to have comparable performance to other molecular biomarkers with published research such as methylation^9^, HPV genotyping^10^ and P16/Ki67 dual-staining^11^, these biomarkers may have the potential to be part of new triage methods for HPV-positive women in the future. miRNAs are highly stable molecules and easily detected by PCR techniques, methods that could be implemented in first points of care in the near future with facilities such as isothermal amplification^12^. In addition, miRNAs as an emerging molecular test could be incorporated in techniques of reflex testing on self-sampling modality to increase the global uptake for triaging hrHPV positive women after primary cervical cancer screening^13^.

The evidence of the role of miRNAs in the development of persistent hrHPV infection and cervical precancerous lesions is emerging. The deregulated expression of hrHPV E6 and E7 genes are the major drivers of uncontrolled cell cycle progression and cervical cancer development^14^. The expression of miRNAs processing proteins, DROSHA and DICER, is increased by hrHPV E6 and E7 proteins^15^ and altered in hrHPV-induced cancer^16^. Furthermore, the modulation of the expression of human miRNAs and the consequent impact on cellular gene expression by hrHPV E6 and E7 contributes to cervical cancer^17,18^.

In this exploratory discovery study, we used the MiSeq platform to sequence QIAseq^®^ miRNA libraries to identify miRNAs differentially expressed between hrHPV-positive-high- (CIN2, CIN3 and SCC; combined as CIN2+) and -low-grade (CIN1, Negative; combined as ≤ CIN1) cervical lesions. Validation of differentially expressed miRNAs was conducted in an independent set of samples by RT-qPCR. Finally, we conducted an analysis to comprehensively capture the potential interaction between differentially expressed miRNAs with mRNA transcripts of proteins of pathways deregulated by HPV E6 and E7 oncoproteins. Our study contributes to elucidating the utility of miRNAs as biomarkers to detect cervical cancer precursor lesions.

## Methods

### Selection of study participants

We studied women selected from the participants of a pragmatic trial (ASCUS-COL trial conducted from 2011 to 2016)^19^ that compared the performance for detection of CIN2+ of strategies that included colposcopy and/or biopsy, repeat cytology, or hrHPV testing for the optimal management of ASC-US (Atypical Squamous Cells of Undetermined Significance) cytology. Another group of participants had been recruited through a screening study^20^, (BIOMARKERS) conducted from 2013 to 2015, that compared the performance for detection of CIN2+ of hrHPV testing, HPV 16/18 genotyping, p16/Ki67 dual-staining, and conventional cytology. Participants (20–69 years old) of these studies were residents of the metropolitan area of Medellin, Colombia, recruited in routine screening (n = 2661) with first-time ASC-US cytology during the last 3 months before recruitment (ASCUS-COL trial) or at colposcopy services (n = 749) with first-time referral to colposcopy because ≥ ASC-US (LSIL, HSIL, ASCUS, ASC-H, AGC) cytology (BIOMARKERS study). Specimens of all women participating in these studies were HPV tested at recruitment by the Hybrid Capture II (ASCUS-COL) or COBAS test (BIOMARKERS study). All participants were followed for at least 24 months or until referred for evaluation by a well-trained colposcopist and biopsied based on the results of the tests (threshold hrHPV-positive and/or ≥ ASC-US cytology) using a standardized and controlled protocol of biopsy sampling. All clinical procedures were conducted under routine care by the local healthcare providers and at the end of the study, accredited experts confirmed histopathological diagnoses. Women with histological diagnoses of CIN2+ by local or expert health providers received treatment. Samples were retrospectively selected, and the selection and laboratory analysis were conducted independently and blindly after the end of the studies. This study was conducted according to the Colombian regulation for research in humans (Resolution 8430 of 1993, Ministry of Health of Colombia), and all methods were performed in accordance with the relevant guidelines and regulations by the institutions.

### Selection of the clinical specimens

**Figure 1:**
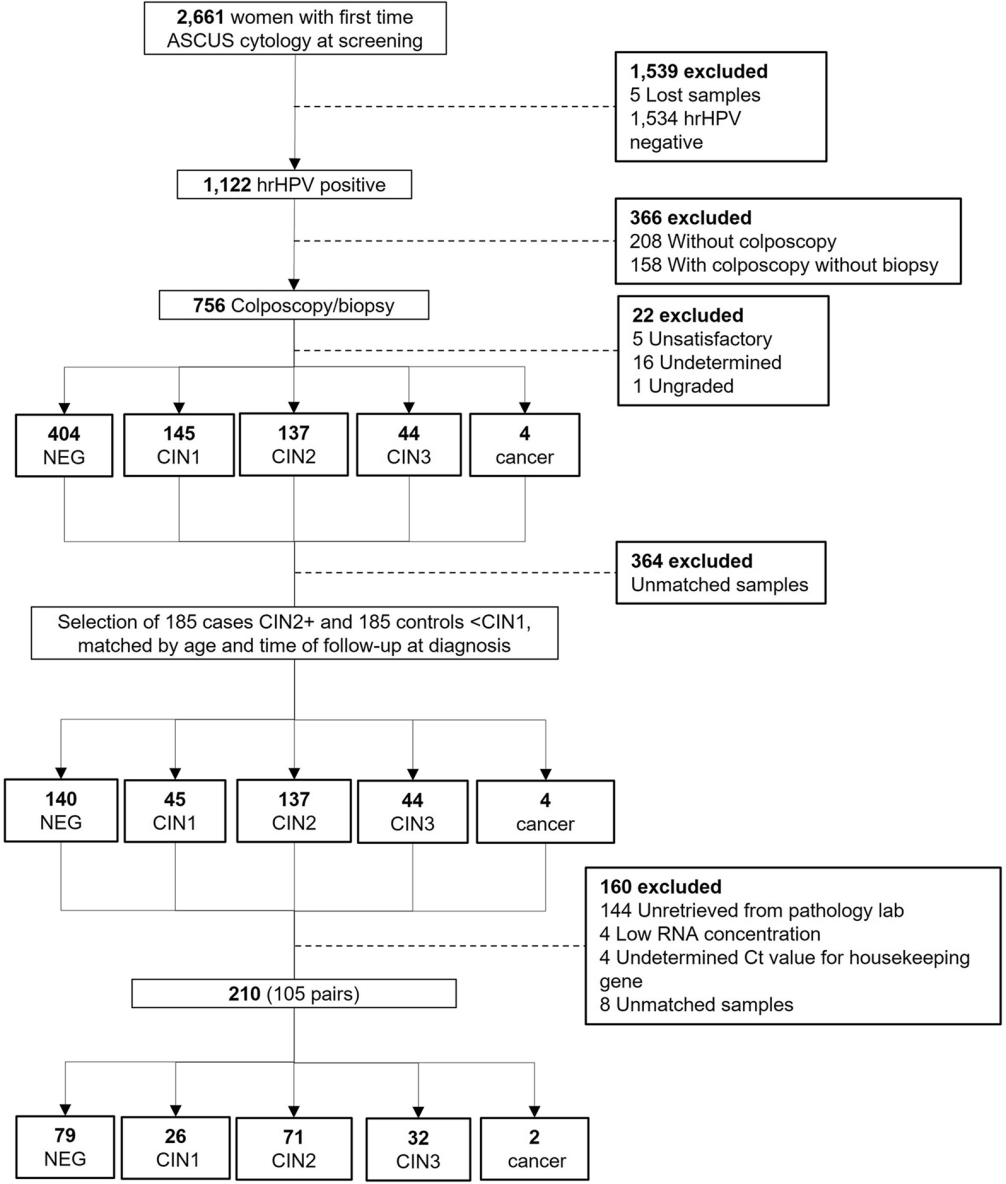
Flowchart of the selection of the validation samples set. *CIN1* cervical intraepithelial neoplasia grade 1, *CIN2* cervical intraepithelial neoplasia grade 2, *CIN3* cervical intraepithelial neoplasia grade 3. Cancer (*SCC* squamous cervical carcinoma).

The clinical specimens used in this study were 10% buffered formalin-fixed and paraffin-embedded (FFPE) tissues identified after the end of the ASCUS-COL or BIOMARKERS studies from women who had hrHPV-positive test results at recruitment visit and with a confirmed colposcopy-directed biopsy with either negative, CIN1, CIN2, CIN3 or carcinoma in situ, adenocarcinoma (ADC) or squamous cell carcinoma (SCC) as the worst diagnosis at any time during the study but before treatment. For the discovery phase, a convenience set of 10 hrHPV-positive FFPE tissue specimens with confirmed histopathology diagnosis of CIN2+ (7 CIN2 and 3 CIN3; median age 32 years; range 22–48) and 10 age-matched, hrHPV-positive FFPE tissues with confirmed histopathology diagnosis of ≤ CIN1 (9 CIN1 and 1 Negative for lesion; median age 29 years; range 22–58), were randomly selected. An independent set of samples was chosen from the ASCUS-COL study to validate by quantitative RT-PCR (qPCR) 5 miRNAs, 2 with the lowest coefficient of variation and 3 with the highest fold change. As shown in the flowchart (Fig. 1), 756 of the 1122 hrHPV positive women had a biopsy, of which 185 corresponded to high-grade preneoplastic cervical lesions (CIN2+), which were selected and paired with women with low-grade lesions (≤ CIN1) by age and time of follow up to diagnosis. Samples from 366 women that did not meet matched criteria or were not randomly selected, 144 women without FFPE tissues in the pathology labs and 16 that did not meet quality criteria for the RT-qPCR were excluded. The independent set included 105 of the 185 randomly selected FFPE tissues of women with the worst biopsy-confirmed diagnosis of CIN2+ (71 CIN2, 32 CIN3 and 2 SCC, median age 29 years; range: 20–60), paired by age (± 6 years) with 105 of the 185 FFPE tissues of women with the worst biopsy-confirmed diagnosis of ≤ CIN1 (26 CIN1, 79 Negative, median age 28 years; range 20–62). HPV genotyping of the samples by CLART HPV4 (Clinical Array Technology, Genomica, Madrid, Spain) conducted at the Queen's Medical Research Institute of The University of Edinburgh (Edinburgh, United Kingdom) is already described^21,22^. Supplementary Tables 1 and 2 show the sociodemographic description of patients of the discovery and validation sets, respectively.

### RNA extraction

Total RNA was isolated using the miRNeasy FFPE Kit (cat 217504, QIAGEN^®^) according to the manufacturer’s instructions. Briefly, the paraffin was removed with Xylene and the samples were incubated at 56 °C for 15 min, then at 80 °C for 15 min in an optimized lysis buffer, which contains Proteinase K for RNA release from tissue sections. The sample was then treated with DNase I to eliminate genomic DNA. The lysate was then mixed with a guanidine salt-based buffer to provide appropriate RNA binding conditions and then ethanol was added, and the samples were transferred to a RNeasy MinElute spin column, where the total RNA binds to the membrane, and contaminants are washed. Finally, the sample containing the total RNA, including miRNAs, was eluted in 20 μL of RNase-free water and stored at -80° C, until its use. The total RNA isolated was quantified using the Qubit™ RNA Assay kit (Invitrogen™), on the Qubit 3.0 Fluorometer (Thermo).

### miRNA library preparation and sequencing

Library preparation was performed from 100 ng of total RNA using the QIAseq^®^ miRNA Library Kit according to the manufacturer’s instructions (cat 331502, QIAGEN^®^). Briefly, a pre-adenylated adapter was ligated to the 3′ end of the miRNAs while an RNA adapter was ligated to the 5′ end of all mature miRNAs. cDNA synthesis was carried out with a reverse transcription primer that binds at the 3′ adapter and incorporates a universal molecular index (UMI). The cDNA was cleaned up using magnetic beads and the library was amplified using a universal forward primer and indexing reverse primers. The libraries were cleaned up with magnetic beads and qualified on an Agilent 2100 Bioanalyzer (Agilent Technologies) using a High Sensitivity DNA 1000 chip, according to the manufacturer’s instructions. The miRNA Library concentration was determined using the Qubit™ dsDNA HS Assay kit (Invitrogen™), on the Qubit 3.0 Fluorometer (Thermo). The libraries were adjusted to 4 nM, pooled (2 10-sample pools) and sequenced at 1 × 75 bp on the MiSeq platform (Illumina, San Diego, CA) following the instructions of the MiSeq System Denature and Dilute Libraries Guide insert.

### miRNA sequencing data processing

FASTQ files generated by the MiSeq instrument were uploaded to GeneGlobe Data Analysis Center of Qiagen^®^ (https://geneglobe.qiagen.com/analyze/) and analyzed under two steps. In brief, the primary quantification consisted in the trimming of the 3′ adapter and low-quality bases using cut-adapt (cutadapt.readthedocs.io/en/stable/guide.html), discarding reads with less than 16 bp insert sequences or less than 10 bp sequences (defective reads), aligning the reads to the reference transcriptome tolerating up to two mismatches (miRBase V.21 http://www.mirbase.org/) using Bowtie (bowtie-bio.sourceforge.net/index.shtml). On average 2.8 million reads per sample were obtained in the sequencing and 1.3 million of these (47% of the reads total) were mapped to miRBase V.21. A total of 2,186 sequences of mature miRNAs were mapped. The secondary analysis consisted of the miRNA differential expression between CIN2+ and ≤ CIN1 groups. The counts were normalized using the DESeq2 scaling factor by the Geometric Median strategy^23^. The differential gene expression analysis was carried out by comparing the miRNAs expression levels between CIN2+ and ≤ CIN1 groups, using Wald statistic test for multiple comparisons, and the p-values adjusted by the Benjamini and Hochberg method (FDR ≤ 0.05). The differential expression analysis was confirmed in R (v3.6.3) using the same DESeq2 workflow. R (v3.6.3) was also used for post-analysis, heatmaps and plots. Raw sequencing reads and normalized reads counts are available from the NCBI Gene Expression Omnibus (GEO) through the series accession number GSE167858.

### Quantitative RT-PCR (qPCR)

The miRCURY Locked Nucleic Acid (LNA) RT Kit (cat 339340; QIAGEN^®^) was used for the miRNA reverse transcription reaction. In brief, total RNA was used as starting material and the procedure involved the miRNA polyadenylation and reverse transcription in a single reaction step using a Poly-T primer bound to a universal tag. Using one universal RT reaction and the cDNA template for subsequent PCR amplifications, the reaction was incubated at 40–42 °C for 1 h, followed by an inactivation step through a brief incubation at 95 °C. The reaction volume for the cDNA synthesis was 10 µL, consisting of 2 µL of total RNA at 5 ng/µL, 2 µL of 5× miRCURY RT Reaction Buffer, 1 µL of 10× miRCURY RT Enzyme mix and 5 µL RNAse-free water. miRCURY LNA SYBER Green PCR Kit (cat 339346; QIAGEN^®^) and miRCURY LNA PCR Assays (YP00204788, YP00205992, YP00204570, YP00204698, YP00204765; QIAGEN^®^) were used for quantitative PCR duplicate reactions per sample. The reference gene used was the small nucleolar RNA (snoRNA) SNORD44 (YP00203902; QIAGEN^®^), widely used as a housekeeping gene in miRNAs studies^24^. Quantitative PCR reactions were performed in 10 μL, consisting of 3 µL of 1:60 diluted cDNA, 5 µL of 2× miRCURY SYBR^®^ Green Master Mix, 1 µL of PCR Primer and 1 µL of RNase free water. Cycle conditions used for cDNA synthesis and PCR were done according to the manufacturer’s protocol. The relative expression was determined by the 2^−ΔCT^ method^25^. The expression of the five miRNAs relative to the internal control gene (SNORD44) in each of the lesion grades (79 NEG, 26 CIN1, 71 CIN2 and 32 CIN3 plus 2 cancers) or grouped as high- (CIN2+) or low-grade (≤ CIN1) lesions were estimated as *∆Ct* = *Ct (miR‑of‑interest) – Ct (SNORD44)* and the median of the Log_2_(2^−∆CT^) of these values plotted in the figures.

### Statistical analysis of miRNA RT-qPCR data

The analysis was based on a prespecified analytical plan. The primary hypothesis was that a sample size of 106 pairs will be needed to estimate, with an 80% power and significance level of 95%, statistical differences between CIN2+ and ≤ CIN1 of a miRNA relative change with a moderate effect (0.3). Based on the distribution of the Ct values for miR-133a-3p, we excluded 2 samples with extreme outliers (Ct values of 14.26 and 8.84). The Cuzick trend test was used to investigate changes in each miRNA with increasing lesion severity. We used the U-Mann Whitney statistic test for the comparison of relative expression medians between CIN2+ and ≤ CIN1 groups. We also developed a miRNA signature, using logistic regression models with CIN2+ or CIN3+ as outcomes and ≤ CIN1 as reference group. Models were run entering all the negative of normalized expression levels relative to SNORD44 (dCt) of the 5 miRNAs and by backward stepwise selection including or not HPV 16 and/or HPV 18 genotypes as a single dichotomous variable. The performance of the most optimal model to predict CIN2+ or CIN3+ was that with the highest AUC and corresponding significant 95% confidence intervals (CI). The value of the 5 miRNAs to triage hrHPV positive women was also evaluated by comparing the performance to detect CIN2+ and CIN3+ with HPV 16/18 genotyping, a marker that has validated clinical utility for the management of hrHPV positive women. A Receiving Operating Characteristics (ROC) analysis including the normalized Ct values of the 5 miRNAs validated by RT-qPCR was conducted to identify the cut-off corresponding to a specificity of 70%. Differences in sensitivities and specificities of the 5 miRNAs signature with HPV16/18 genotypes or by combining both tests were examined by McNemar’s test with continuity correction. Age is one of the most important risk factors for cancer overall^26^; therefore, if the miRNAs expression is associated with preneoplastic high-grade lesion and cancer, it can also be associated with older age. We observed that regardless of the histological diagnosis, the relative expression of miR-133a-3p was different in women ≤ 30 as compared to women > 30 years old (p = 0.009) (Supplementary Fig. 1). Therefore, we only included in the final RT-qPCR validation analysis samples from women with their respective paired control (+ /− 6 years, n = 210) (see flowchart, Fig. 1). For all tests, a p-value ≤ 0.05 was considered statistically significant. The software R (v3.6.3) was used to conduct the analysis.

### Cellular miRNAs interaction with proteins of the pathways altered by HPV E6/E7 oncoproteins (in-silico approach)

To explore the potential for functional importance of the miRNAs in the context of HPV infection, we searched for experimentally proven inverse correlations between the miRNAs and their corresponding target genes products using the Ingenuity Pathway Analysis (IPA, QIAGEN Inc., https://digitalinsights.qiagen.com/IPA)^27^. IPA integrates the data from the databases TargetScan, TarBase, miRecords and Ingenuity Expert Findings. The names of the top 25 differentially expressed miRNAs between CIN2+ and < CIN1 (p value < 0.05), were uploaded to IPA. This list of mRNAs, was subsequently filtered using the following criteria: selecting only “Experimentally observed” in the column “miRNA confidence”, “Cancer” in the column “Disease”, “Human” in the column “Species” , and finally filtering out genes without biological relevance in the column “Pathway”. Thereafter, the information from the KEGG PATHWAY ko05165 for Human Papillomavirus infection (https://www.genome.jp/pathway/map05165+K04693) was used to predict miRNAs with potential interactions with gene target products of pathways that are altered by HPV E6 and/or E7 oncoproteins^28–30^. The interactions between miRNAs and mRNAs related to HPV infection was built using Cytoscape V3.7.1, (URL: https://cytoscape.org/)^31^.

### Ethical considerations

All participants included in the ASCUS-COL and BIOMARKERS studies signed informed consent and authorization to use samples and data for future research. The ethics committees for human experimentation of Sede de Investigación Universitaria (SIU) (Resolutions 08-036-171 and 18-40-535) and School of Medicine (Resolution 004/2008), of the Universidad de Antioquia approved the studies.

## Results

### miRNA signatures on high- vs. low-grade cervical lesions

There were no differentially expressed miRNAs between the samples from the ASCUS-COL and the BIOMARKERS studies (data not shown). The analysis of the sequencing data (discovery set) showed 162 miRNAs differentially expressed between CIN2+ and ≤ CIN1 lesions (p ≤ 0.05). The top 25 miRNAs differentially expressed are shown in Table 1, and the complete list of differentially expressed miRNAs is shown in Supplementary Table 3.

**Table 1:** Fold changes and coefficient variations of the difference of normalized counts of the top 25 differentially expressed miRNAs identified by miRNAseq between CIN2+ or CIN3+ and the reference group (< CIN1).

miRNA	CIN2+ (n = 10)		CIN3 (n = 3)		% CV
	FC	p-value	FC	p-value	
**miR-133a-3p**	**6.9**	** < 0.001**	**3.5**	**0.001**	**44**
**miR-143-5p**	**5**	** < 0.001**	**2.7**	**0.008**	**30**
**miR-143-3p**	**5.1**	** < 0.001**	**2.6**	**0.011**	**19**
miR-145-5p	4.8	< 0.001	2.5	0.018	22
miR-145-3p	4.5	< 0.001	2.3	0.032	30
miR-1-3p	4.4	< 0.001	3.1	0.002	48
miR-9-3p	3.3	< 0.001	1.9	0.079	26
miR-99a-3p	3.4	0.001	1.9	0.096	25
miR-376b-3p	3.8	0.001	2	0.069	36
miR-125b-2-3p	2.8	0.001	1.7	0.145	21
let-7c-5p	3	0.001	1.9	0.095	13
miR-4329	2.4	0.002	1.6	0.149	18
**miR-29a-3p**	**2.6**	**0.002**	**1.7**	**0.137**	**10**
miR-28-3p	2.5	0.002	1.9	0.066	13
miR-125b-5p	2.9	0.002	1.7	0.158	12
miR-199a-5p	3.1	0.002	1.6	0.214	18
miR-9-5p	3.2	0.002	1.7	0.15	25
miR-214-5p	3.1	0.002	1.6	0.205	29
miR-619-3p	2.1	0.002	1.4	0.281	15
miR-381-3p	3.2	0.002	1.7	0.144	28
miR-195-3p	2.9	0.002	1.6	0.197	27
**miR-30b-5p**	**2.1**	**0.002**	**1.7**	**0.085**	**9**
miR-99a-5p	2.8	0.003	1.6	0.184	14
miR-204-5p	2.9	0.003	1.9	0.089	25
miR-10b-3p	2.4	0.003	1.8	0.111	19

The p-values attained by the Wald statistic test are corrected for multiple testing using the Benjamini and Hochberg method by default in the DESeq2 package in use at the Data Analysis Center (Qiagen); *CV* coefficient of variation (n = 20 samples) of the normalized counts for each miRNA. miRNAs bolded were validated by qRT-PCR. *FC* fold change (CIN2+/ ≤ CIN1), or (only CIN3/≤ CIN1).

**Figure 2:**
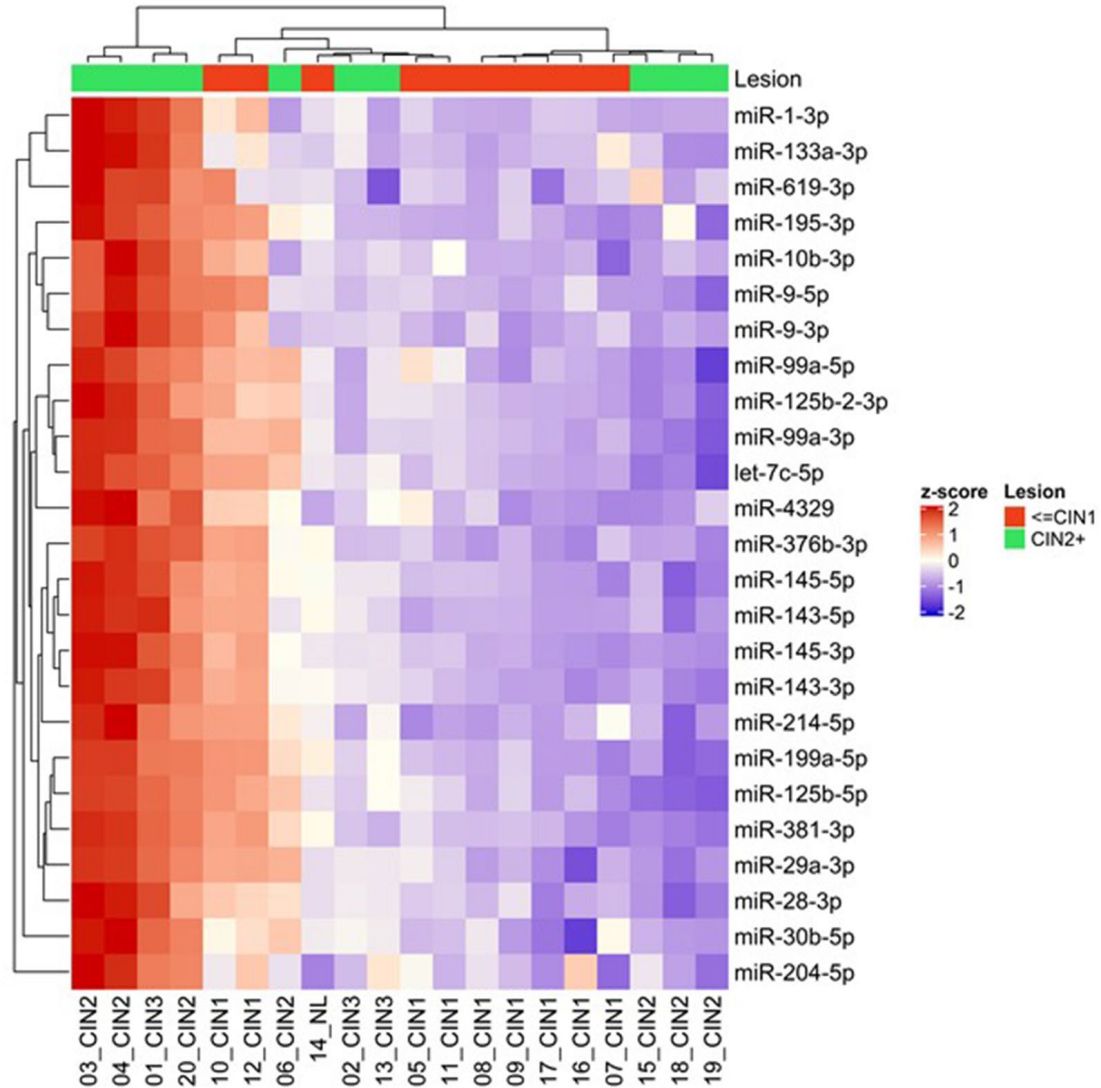
Heat map of differentially expressed miRNAs (Top 25) with statistical significance (p < 0.05) which were increased in CIN2+ respect to ≤ CIN1, using differential expression analysis in DESeq2. The data correspond to the normalized counts to the Z-score (math transformation). Heatmaps were generated with normalized counts to the Z-score (math transformation) using the package ‘pheatmap’ using R version 1.0.12 (https://cran.r-project.org/web/packages/pheatmap/index.html).

Unsupervised hierarchical clustering was performed for the 25 most differentially expressed miRNAs between the 10 CIN2+ and 10 ≤ CIN1 lesions, using the z-score transformation on the normalized read counts. The heat map shows that, compared to the ≤ CIN1 lesions which tend to group in the center of the heat map, the CIN2+ lesions are distributed in three different clusters, suggesting a higher miRNA expression variability in CIN2+ as compared to the ≤ CIN1 lesions (Fig. 2). The higher variability in high-grade, as compared to low-grade cervical lesions was observed for all the 162 differentially expressed miRNAs across the 20 samples (Supplementary Fig. 2).

**Figure 3:**
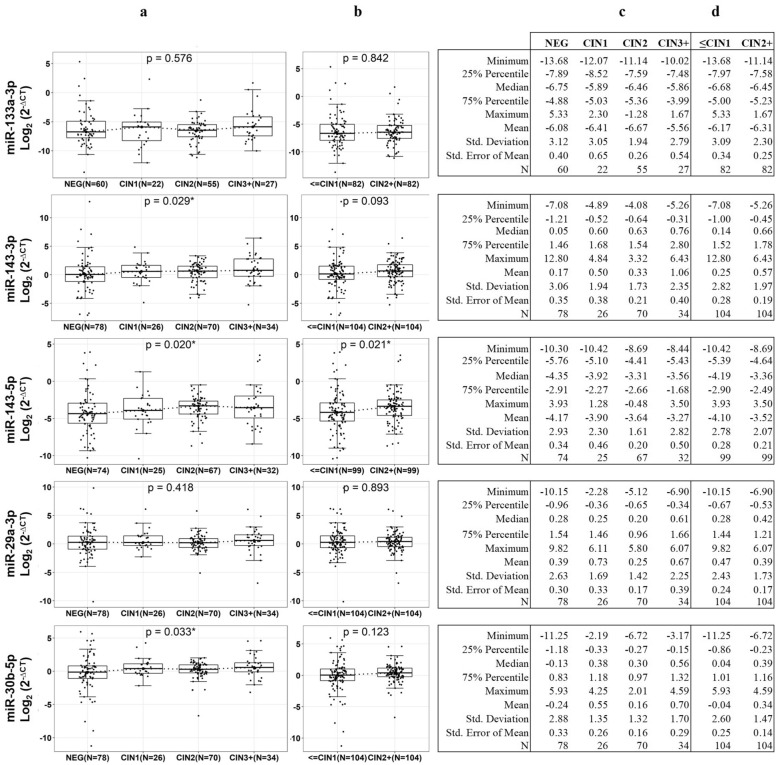
Relative expression levels (log_2_(2^−∆CT^)) of five miRNAs in the validation set of samples in every histopathology lesion grade (NEG, CIN1, CIN2 and CIN3+) **(a)**, and in ≤ CIN1 and CIN2+ **(b)**. Relative expression was determined by 2^−∆CT^ method by normalizing the Ct values, estimating the difference between the Ct value of each miRNA and the housekeeping gene (*SNORD44*) in each sample. The horizontal bars represent the median and the whiskers represent the highest and lowest point within 1.5 times the interquartile range. The values outside the whiskers are outliers. Cuzick trend test (p < 0.05) was used to investigate continuous changes in relative expression with increasing lesion severity, and Mann Whitney test to compare medians between high- and low cervical lesions. Descriptive statistics of the relative expression levels (log_2_(2^−∆CT^)) of the five miRNAs in the validation set of samples in every histopathology lesion grade (NEG, CIN1, CIN2 and CIN3+) **(c)**, and in ≤ CIN1 and CIN2 + **(d)**.

We selected three miRNAs with the highest FC (miR-133a-3p, miR-143-5p, miR-143-3p) and two miRNAs with the lowest %CV (miR- 29a-3p and miR-30b-5p) from the top 25 of the 162 miRNAs differentially expressed for validation on an independent group of FFPE tissues (n = 210, 105 CIN2+ lesions matched by age and time to diagnosis to 105 ≤ CIN1 lesions). Undetermined Ct values occurred in 21 samples for miR-133a-3p, in 6 samples for miR-143-5p, and in 1 sample for miR-29a-3p, miR-30b-5p, and miR-143-3p. Therefore, their age-paired samples were also excluded from this analysis (see flowchart, Fig. 1). The summary of RT-qPCR data is shown in Supplementary Table 4. The variability of the five miRNAs used in qPCR validations was also observed in the RNAseq data. We conducted linear regression and observed a linear relationship (Coefficient of determination R^2^) between the median (R^2^ = 0.90) or mean (R^2^ = 0.88) of normalized read-counts of RNAseq and normalized Ct values of RT-qPCR (Supplementary Fig. 3). We observed a significant trend for the medians (Cuzick test for medians trend, p < 0.05) of the relative expression of miR-143-3p (p = 0.029), miR-143-5p (p = 0.020) and miR-30b-5p (p = 0.033) with increasing severity of the lesions (Fig. 3a). When we compared the relative expression levels between high-grade lesions (CIN2+) and low-grade lesions (≤ CIN1), we observed a significant increased relative expression of miR-143-5p in the CIN2+ lesions (Fig. 3b, p = 0.021), the descriptive statistics across the cervical lesions, and between CIN2+ and ≤ CIN1 for each miRNA are shown in (Fig. 3c) and (Fig. 3d), respectively. We did not observe any additional change of the relative expression levels in the other miRNAs.

### Comparison of the performance of the 5 miRNAs signature with HPV16/18 genotyping to detect CIN2+ or CIN3+ in HPV positive women

The AUCs of the models for CIN2+ or CIN3+ that included all five miRNAs were 0.645 (95% CI 0.565–0.719, p < 0.001) and 0.649 (95% CI 0.551–0.739, p < 0.011) respectively. The best performer predictor signature was observed when HPV 16 and/or 18 genotypes were added as a single dichotomous variable to the five miRNAs classification with AUCs of 0.678 (95% CI 0.596–0.751, p < 0.001) for CIN2+ and 0.726 (95% CI 0.628–0.811, p < 0.001) for CIN3+ (Table 2). Supplementary Table 5 shows the number and percentages of  ≤ CIN1 controls and CIN2+ and CIN3+ cases with negative or positive tests results. Supplementary Table 6 shows the comparison of the performance of the five miRNAs signature, HPV 16/18 genotyping or the combination of both to detect CIN2+ and CIN3+ at a desired specificity of 70.88% (cut-off for the 5 miRNAs signature ≥ 0.54). The specificity of HPV 16/18 genotyping (65.28%, 95%CI 53.14–76.12) was not statistically different to that of the 5 miRNAs signature at a cut-off of ≥ 0.54 for a set specificity of 70.88% (95%CI 59.58–80.57). Likewise, there were not differences in the performance for the detection of CIN2+ or CIN3+ between the five miRNAs signature and HPV 16/18 genotyping. However, compared to the five miRNAs signature, the specificity for ≤ CIN1 significantly decreased (p = 0.0001) from 70.88 to 49.73% (95% CI 42.48–56.97) and the sensitivity significantly (p < 0.0001) increased from 51.90% (95% CI 40.36–63.30) to 77.22% (95% CI 66.40–85.90) for detection of CIN2+ and from 51.85% (95% CI 31.95–71.33) to 85.19% (95% CI 66.27–95.81) for detection of CIN3+ (p = 0.0076) after combining the positivity for any of the two, the five miRNAs signature plus HPV 16/18 genotyping.

**Table 2:** Multivariate logistic regression analysis for CIN2+ and CIN3+ prediction by miRNAs only or in combination with HPV 16 and/or HPV 18 genotypes.

Variable selection method	miRNA	CIN2+ (n = 79) ref. ≤ CIN1 (n = 79)								CIN3+ (n = 27) ref. ≤ CIN1 (n = 79)							
		p^a^	OR	95% CI		AUC	95% CI		p^b^	p^a^	OR	95% CI		AUC	95% CI		p^b^
Enter	miR-143-5p	**0.018**	1.357	**1.055**	**1.747**	0.645	0.565	0.719	** < 0.001**	0.410	1.162	0.813	1.661	0.649	0.551	0.739	**0.011**
	miR-29a-3p	0.129	0.825	0.644	1.058					0.321	0.855	0.627	1.165				
	miR-133a-3p	0.264	0.900	0.749	1.083					0.745	0.959	0.745	1.235				
	miR-143-3p	0.630	0.942	0.739	1.201					0.515	1.132	0.779	1.646				
	miR-30b-5p	0.608	1.072	0.822	1.399					0.622	1.091	0.770	1.547				
Backward	miR-143-5p	**0.015**	1.235	1.041	1.464	0.613	0.533	0.690	**0.007**	0.074	1.155	0.986	1.353	0.621	0.522	0.713	**0.061**
	miR-29a-3p	**0.049**	0.820	0.673	0.999					–	–	–	–				

		CIN2 + (n = 78) ref. ≤ CIN1 (n = 72)								CIN3 + (n = 27) ref. ≤ CIN1 (n = 72)							
Enter	miR-143-5p	**0.020**	1.355	1.048	1.751	0.678	0.596	0.751	** < 0.001**	0.179	1.196	0.837	1.833	0.726	0.628	0.811	** < 0.001**
	miR-29a-3p	**0.048**	0.749	0.563	0.997					0.214	0.803	0.568	1.135				
	miR-133a-3p	0.378	0.918	0.758	1.111					0.804	0.968	0.746	1.256				
	miR-143-3p	0.836	1.028	0.791	1.336					0.362	1.212	0.801	1.833				
	miR-30b-5p	0.728	1.051	0.793	1.393					0.734	1.065	0.739	1.535				
	HPV16 and/or18	**0.035**	2.097	1.054	4.173					**0.023**	3.068	1.171	8.039				
Backward	miR-143-5p	**0.004**	1.313	1.093	1.578	0.660	0.578	0.735	** < 0.001**	**0.036**	1.240	1.015	1.528	0.704	0.604	0.791	** < 0.001**
	miR-29a-3p	**0.016**	0.752	0.597	0.947					–	**–**	–	–				
	HPV16 and/or18	**0.031**	2.123	1.071	4.209					**0.022**	3.015	1.172	7.757				

Significant p values (< 0.05) in bold.

^**a**^p-value for prediction of the outcomes of the 5 miRNAs signature only or in combination with HPV 16 and/or HPV 18.

^**b**^p-value to tests the probability that the observed sample Area Under the Curve (AUC) curve is 0.5.

### Role of miRNAs differentially expressed in the context of HPV infection

**Figure 4:**
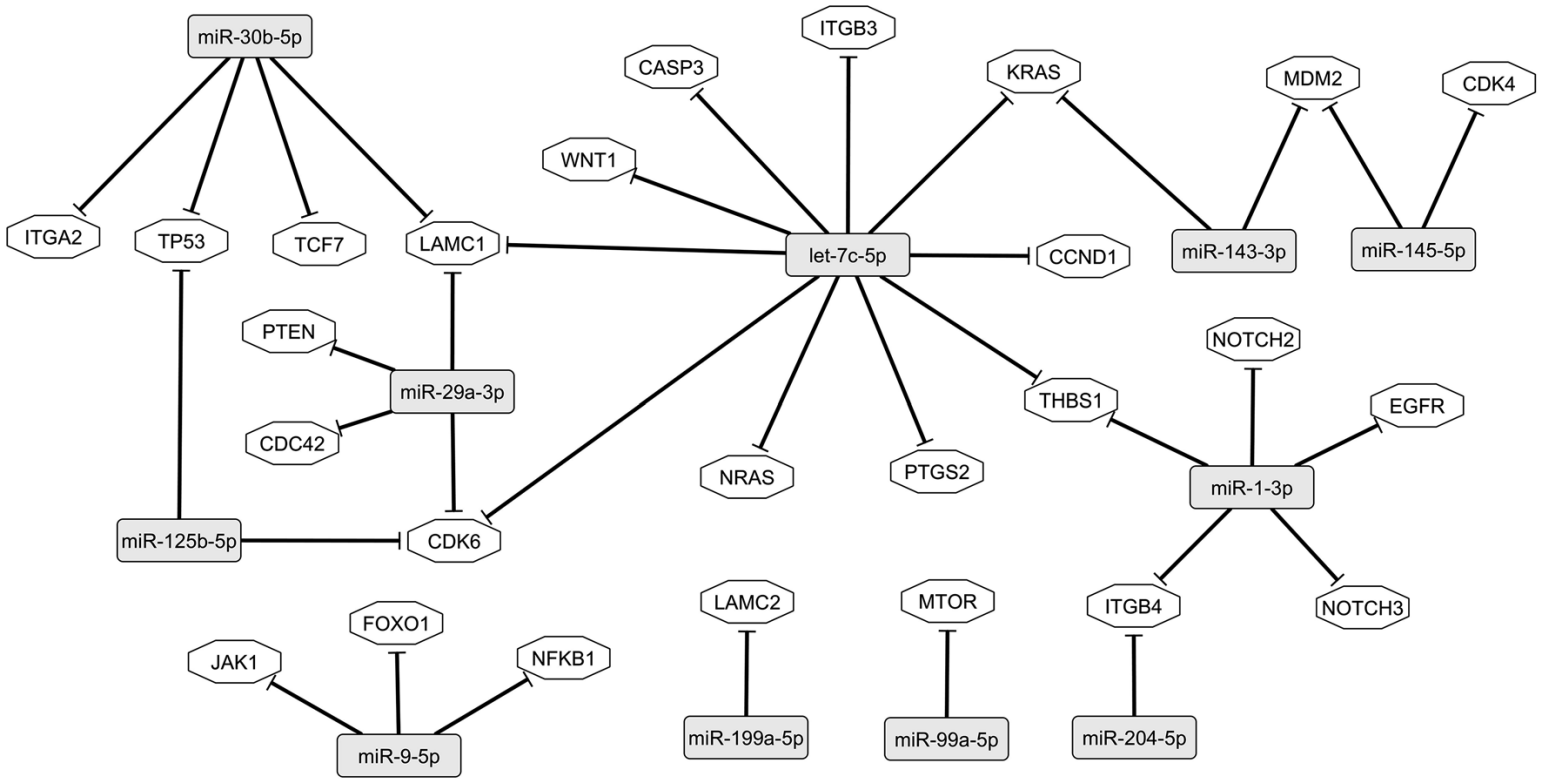
Interaction pathway between differentially expressed miRNAs and their respective inverse correlated mRNAs observed in the Ingenuity Pathway Analysis of proteins of pathways deregulated by HPV E6 and E7 oncoproteins. Twentysix target gene products with inverse correlation with 11 differentially expressed miRNAs were identified in the Ingenuity Pathway Analysis (QIAGEN Inc., https://digitalinsights.qiagen.com/IPA). Proteins of pathways deregulated by HPV E6 and E7 oncoproteins are from the KEGG Human Papillomavirus Infection (https://www.genome.jp/pathway/ko051659). The interactions between miRNAs and mRNAs was built using Cytoscape V3.7.1 (URL: https://cytoscape.org/).

Table supplementary 7 shows miRNAs-target gene products pairs with validated inverse correlations in the IPA. Fourteen out of the top 25 differentially expressed miRNAs have experimental evidence of inverse correlation with 401 unique different target mRNAs. Eleven of these 14, have experimental evidence of targeting 26 proteins of pathways altered by HPV E6 and E7 oncoproteins (Fig. 4) and two of these 11 miRNAs (miR-143-3p and miR-29a-3p) were the best predictors of CIN2+ and CIN3+ when validated in the independent set of samples by RT-qPCR (Fig. 4 and Table 2).

## Discussion

It has been shown that miRNAs modulate the expression of tumor suppressor genes related to the hrHPV carcinogenic processes such as *TP53*, *CDKN1A* (p21) and *CDKN1B* (p27)^32,33^. The levels of these miRNAs are modulated by hrHPV E6/E7 expression in-vitro, suggesting that miRNAs mediate hrHPV-induced cervical carcinogenesis^17,34^. In this work, we sequenced miRNAs from 20 FFPE tissues from hrHPV positive women with high- or low-grade cervical lesions and identified a panel of 162 miRNAs differentially expressed between those groups using DESeq2^23^. Our screening approach was validated by RT-qPCR in an independent set of 210 FFPE tissue samples (79 NEG, 26 CIN1, 71 CIN2, 32 CIN3, and 2 SCC). Finally, the in-silico exploration showed that the miRNAs validated in this study interact with genes coding for proteins of pathways altered by HPV E6/E7 oncoproteins. The Ingenuity Pathway Analysis identified 401 unique different inverse correlated target mRNAs for 14 out of the top 25 differentially expressed miRNAs (Supplementary Table 7). Eleven of these 14 miRNAs were predicted to interact with 26 proteins of pathways altered by HPV E6 and E7 oncoproteins (Fig. 4). Among these 11, miR-29a-3p and miR-143-3p, which respectively interact with the important tumor suppressor genes PTEN, and KRAS, were the best predictors of CIN2+ or CIN3+ in the regression models that included the five miRNAs validated by RT-qPCR in the independent set of samples (Table 2). At a desired specificity of 70.88% (cut-off for the five miRNAs signature ≥ 0.54 normalized counts) the performance for the detection of CIN2+ or CIN3+ of the 5 miRNAs signature was similar to that of HPV 16/18 genotyping. However, compared to the five miRNAs signature, a significant decrease in specificity for ≤ CIN1 and a significant (p < 0.0001) increased in the sensitivity for CIN2+ or CIN3+ was observed after combining the positivity for any of the two, the five miRNAs signature plus HPV 16/18 genotyping. This prompted us to enquire about the proportion of samples positive for the five miRNAs signature among those positive for HPV16/18 or for the other high-risk genotypes. The proportions among the positive for genotypes other than HPV16/18, were 30%, 56% and 64% in ≤ CIN1, CIN2+ or CIN3+ respectively, meanwhile the respective percentages were 32%, 50% and 44% in those HPV16/18 positive. This indicates that miRNAs detected an additional 20% of CIN3+ cases not detected by HPV16/18 genotyping, an attribute important for the design of algorithms including reflex management of other than hrHPV 16/18 positive women.

Deep sequencing analysis of small RNA molecules is a powerful technique to obtain detailed information of the miRNAs profiles of a biological sample^35^. To the best of our knowledge, ours is the first study to determine genome-wide miRNA profiles from hrHPV-positive FFPE cervical tissue with preneoplastic lesions using deep sequencing analysis. One of the strengths of our study is the inclusion of patients with histologically confirmed FFPE tissues. By inclusion criteria only women 20–69 years old women (birth years 1942–1993) were included in the cohort where this study was nested. Women were recruited between January 2011 and January 2014. HPV vaccine was introduced for 2003 birth cohorts (9 years old girls only) in Colombia in 2012. Therefore, participants in this study were not eligible for vaccination. Indirect evidence of low probability of vaccination of this cohort is the fact that HPV infection rates were 40%, which is the expected rate for women with ASC-US cytology at this age range^19^. Our study had the representation of the complete spectrum of cervical lesions, which are necessary for a complete understanding of the miRNAs profiling on each stage. Another strength of our study is that it uses a protocol for small RNA library construction, optimized with Unique Molecular Indexes (UMIs) (a twelve-bases random sequence) enabling unbiased and accurate miRNome-wide quantification of mature miRNAs by NGS. The UMIs allow the tagging of each mature miRNA molecule, removing any sequencing bias introduced during library construction due to different amplification efficiencies^36^. One interesting observation was that the age of the participants influenced the relative expression of miRNAs based on the aggressiveness of the preneoplastic lesions of the cervix (Supplementary Fig. 1). We found that age could be a confounding variable in the expression of miR-133a-3p and miR-30b-5p. The relative change of miR-133a-3p was found to be higher in older women (> 30 years old) when compared to younger women ≤ 30 years old (p = 0.009). The relative change of miR-30b-5p was higher in older (> 30) than in younger women with CIN2 (p = 0.039). These observations guided our decision to pair age-matched participants to compare high- (CIN2+) vs. low-grade lesions (≤ CIN1) for our validation set.

Snoek et al. conducted a discovery analysis in 56 hrHPV-positive cervical self-collected samples (32 ≤ CIN1 and 24 CIN3) by small RNAseq (Illumina, Inc, San Diego, USA) and logistic regression for identification of differentially expressed miRNAs with validation by RT-qPCR in an independent set of 190 samples (101 ≤ CIN1, 48 CIN3, and 41 SCC)^37^. We did not find commonly differentially expressed miRNAs, probably because of differences in the platforms used, the sample type, and analysis strategy. However, when we analyzed Snoek *et. al.* data available in the Gene Expression Omnibus (GEO number GSE104758) using our strategy, seven of the miRNAs were shared between the two studies (hsa-mir-9-5p, hsa-mir-195-5p, hsa-mir-196b-5p, hsa-mir-504-5p, hsa-mir-30e-3p, hsa-mir-184, hsa-mir-126-5p).

We acknowledge that it would be desirable to confirm in the same set of samples the differential expression of the five miRNAs included in the RT-qPCR. However, in spite of the high variability and small sample size of the RT-qPCR data, there was significant trend of higher expression with increasing lesion severity, in three of the five miRNAs, although this difference was significant for miR-143-5p only. A priori sample size of 106 pairs was estimated to achieve 80% power to compare medians between ≤ CIN1 and CIN2 + lesions (U-Mann–Whitney test), with 1 tail, an effect size of 0.3, at 95% significance level. However, in the post-hoc analysis with an observed effect size of 0.2 for miR-143-5p, a 54% power is reached with the 99 sample pairs analyzed.

Our *in-silico* approach suggests that the miRNAs differentially expressed potentially regulate the expression of genes encoding for proteins of pathways altered by hrHPV E6/E7 oncoproteins. This observation strengths the biological relevance of the miRNAs for distinguish high- from low-grade cervical precancerous lesions. However, it is important to clarify that in-vitro assays are necessary to confirm the role of these miRNAs in hrHPV-induced carcinogenesis. Studies with larger number of samples independently validated by RT-qPCR and correlation analysis between differentially expressed miRNAs and their target pairs using cervical precancerous and cancer lesions and comparison with other biomarkers such as HPV genotyping, methylation and p16/Ki67 dual-staining are also needed to further improve the knowledge about their biological role and clinical utility of these biomarkers to triage hrHPV-positive women. The observation that high variability of miRNA expression is higher in high-grade cervical lesions, suggests that several combined miRNAs may be more suitable as biomarkers for pre-cancerous cervical lesions. The use of cervical exfoliates, in addition to FFPE samples, can improve the performance of the RT-qPCR and therefore capture larger differences in the fold change when the effect size is small.

## Conclusions

In conclusion, a comprehensive analysis of (i) the RNA-Seq data of 10 CIN2+ and 10 ≤ CIN1 precancerous lesions identified 162 differentially expressed miRNAs, (ii) the Ingenuity Pathway Analysis of the top 25 differentially expressed miRNAs identified inverse correlations with 401 unique coded target genes for 14 miRNAs, of which 11 interact with proteins regulated by HPV E6 and E7 oncoproteins and (iii) independent validation of five of these miRNAs by RT-qPCR in 79 CIN2+ and 79 ≤ CIN1 precancerous lesions identified two miRNAs predictors of CIN2+ and CIN3+ precancerous cervical lesions. Our results suggested that these two miRNAs are potential biomarkers for predicting cervical precancerous lesions. However, larger prospective studies are necessary to determine the clinical value of these miRNAs and further experimental validation is required to demonstrate the biological role of them in HPV-related cervical carcinogenesis.

## Supplementary Information

Supplementary Figure 1.

Supplementary Figure 2.

Supplementary Figure 3.

Supplementary Table 1.

Supplementary Table 2.

Supplementary Table 3.

Supplementary Table 4.

Supplementary Table 5.

Supplementary Table 6.

Supplementary Table 7.

## Data Availability

The dataset excluding personal identifiers will be available to proper academic parties on request from the corresponding author in accordance with the data sharing policies of Universidad de Antioquia. Raw sequencing reads and normalized reads counts are available from the NCBI Gene Expression Omnibus (GEO) through the series accession number GSE167858.
