# Supplementary figures and images for: miRNAs signature as potential biomarkers for cervical precancerous lesions in human papillomavirus positive women
Source: Sci Rep. 2023 Jun 17;13:9822. doi: 10.1038/s41598-023-36421-9 (PMC10276834; doi:10.1038/s41598-023-36421-9)

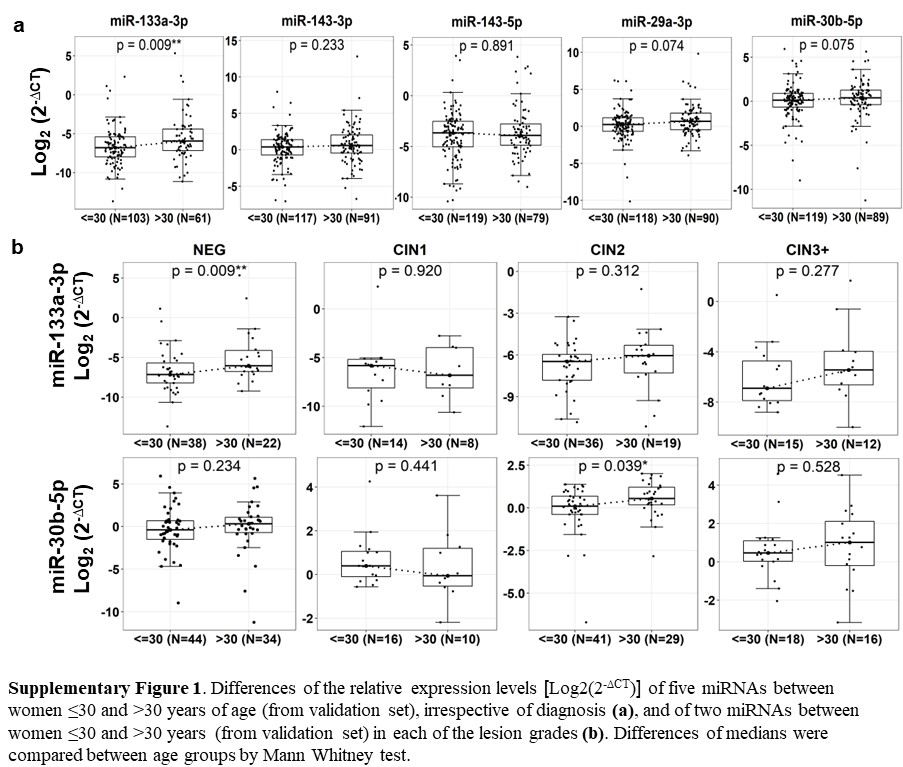

Supplement: Supplementary file 1 — Supplementary Figure 1. [file 41598_2023_36421_MOESM1_ESM.jpg]

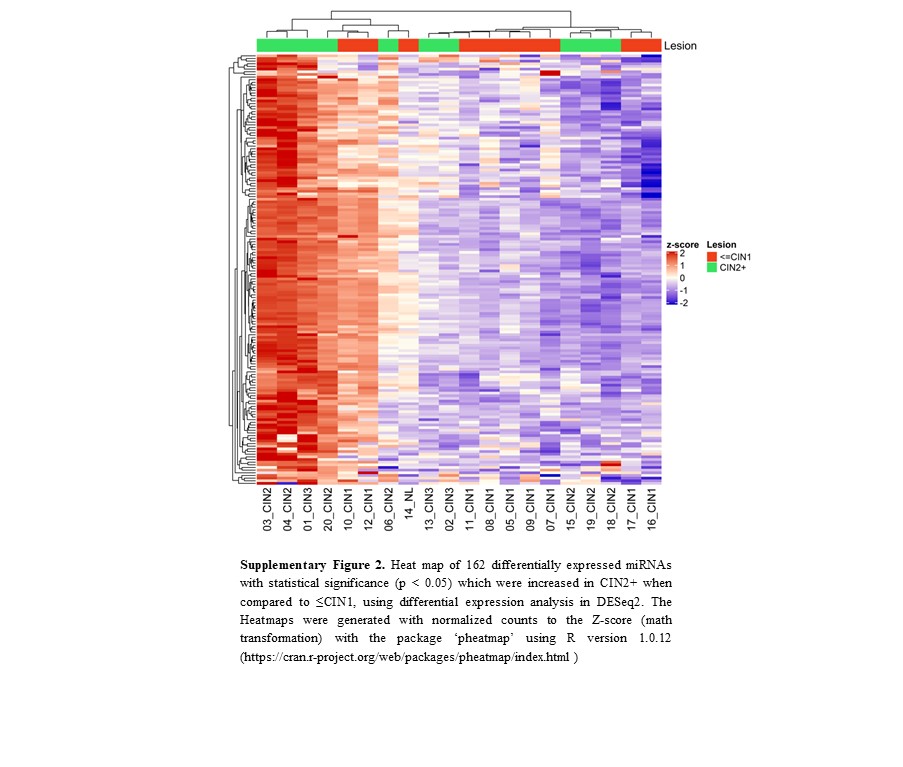

Supplement: Supplementary file 2 — Supplementary Figure 2. [file 41598_2023_36421_MOESM2_ESM.jpg]

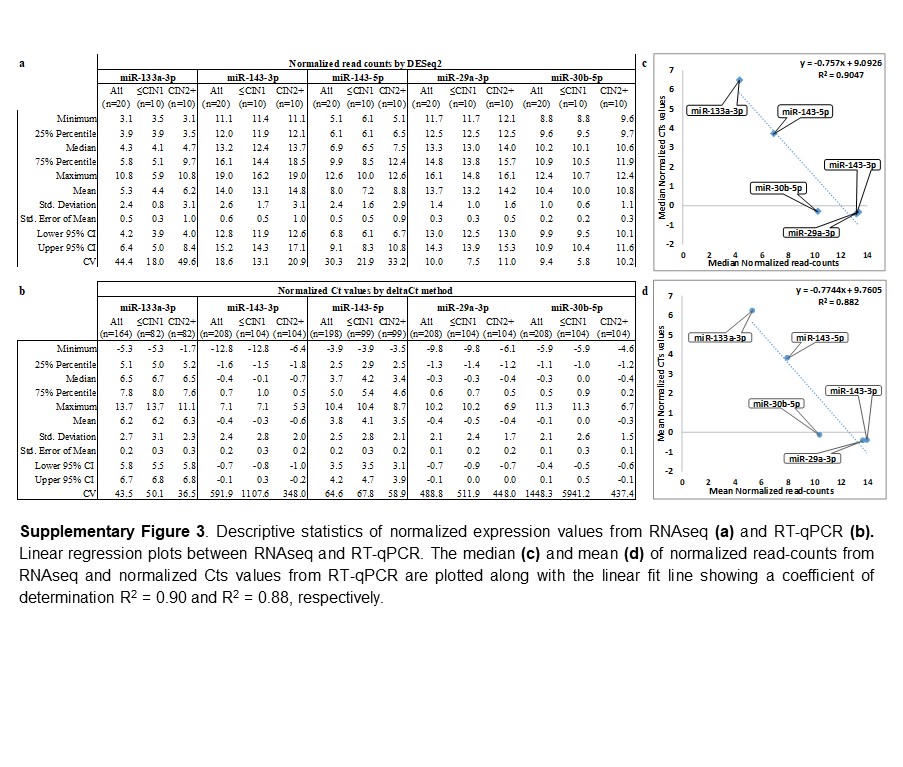

Supplement: Supplementary file 3 — Supplementary Figure 3. [file 41598_2023_36421_MOESM3_ESM.jpg]
