# Supplementary material for: miRNAs signature as potential biomarkers for cervical precancerous lesions in human papillomavirus positive women
Source: Sci Rep. 2023 Jun 17;13:9822. doi: 10.1038/s41598-023-36421-9 (PMC10276834; doi:10.1038/s41598-023-36421-9)
Supplement: Supplementary file 4 — Supplementary Table 1. [file 41598_2023_36421_MOESM4_ESM.pdf]

Supplementary Table 1. Distribution of sociodemographic characteristics and risk factors of study population of the discovery set.

Characteristic	All		≤CIN1		CIN2+		p value[a]
	n = 20	%	n = 10	%	n = 10	%	
NEG	1	(5)	1	(10)	0	(0)	-
CIN1	9	(45)	9	(90)	0	(0)	
CIN2	7	(35)	0	(0)	7	(70)	
CIN3	3	(15)	0	(0)	3	(30)	
**Age (years)**							
Median [IQR]	29.0 [26.5-47.3]		29.0 [26.5-47.3]		32.0 [30.75-38.5]		
≤30	8		6		2		0.068
>30	12		4		8		
**Marital status**							
Divorced/separated/Single	6	(30)	3	(30)	3	(30)	1
Married/cohabiting	14	(70)	7	(70)	7	(70)	
**Education level**							
High School/College or higher	10	(50)	6	(60)	4	(40)	0.371
Up to some/Incomplete High School	10	(50)	4	(40)	6	(60)	
**Social stratum**							
Stratum 1 or 2	10	(50)	6	(60)	7	(70)	0.639
Stratum 3 or more	10	(50)	4	(40)	3	(30)	
**Age of first sexual intercourse (years)**							
Median [IQR]	16.0 [15.0-18.0]		16.0 [15.0-18.75]		16.5 [15.0-18.5]		
≤16	12		7		5		0.361
>16	8		3		5		
**Lifetime sexual partners**							
Median [IQR]	3 [1-4]		2 [1 - 3]		3 [2.5-8.0]		
1 - 2	8		6		2		0.148
3 or more	11		4		7		
Do not know/ No answer	1		0		1		
**Parity**							
Median [IQR]	2 [1-3]		2 [1.75-3.0]		2 [0.75-3.0]		
Never	3		1		2		0.788
1 - 2	10		5		5		
≥3	7		4		3		
**Oral contraceptive intake (years)**							
Median [IQR]	7 [1.15-9.75]		4.1[1.0-9.25]		6.88 [2.5-11.44]		
Never	1		0		1		0.270
<5	7		5		2		
≥5	12		5		7		
**Frequency of cytology use**							
Once or more than once every year	12	(60)	6	(60)	6	(60)	1
Once every 2-10 years	8	(40)	4	(40)	4	(40)	

Abbreviations: CIN: Cervical Intraepithelial Neoplasia. ≤CIN1: Includes biopsy negative lesion (NEG) or CIN1; CIN2+: High-grade neoplasia (CIN2 and CIN3); CIN2: Cervical intraepithelial neopasia grade 2; CIN3: Cervical intraepithelial neoplasia grade 3; RIC: Interquartile range.
[a]Person's chi-squared test.
