# Supplementary material for: miRNAs signature as potential biomarkers for cervical precancerous lesions in human papillomavirus positive women
Source: Sci Rep. 2023 Jun 17;13:9822. doi: 10.1038/s41598-023-36421-9 (PMC10276834; doi:10.1038/s41598-023-36421-9)
Supplement: Supplementary file 5 — Supplementary Table 2. [file 41598_2023_36421_MOESM5_ESM.pdf]

Supplementary Table 2. Distribution of sociodemographic characteristics and risk factors of study population of the validation set**.**

Characteristic	All		≤CIN1		CIN2+		p value[a]	CIN3+		p value[b]
	n = 210	%	n = 105	%	n = 105	%		n = 34	%	
NEG	79	(37.62)	79	(75.24)	0	(0)		0	(0)	
CIN1	26	(12.38)	26	(24.76)	0	(0)		0	(0)	
CIN2	71	(33.81)	0	(0)	71	(67.62)		0	(0)	
CIN3	32	(15.24)	0	(0)	32	(30.48)		32	(94.12)	
SCC	2	(0.95)	0	(0)	2	(1.9)		2	(5.88)	
**Age (years)**										
Median [IQR]	29 [24 - 37]		28 [24 - 37]		28 [24 - 37]			30 [25 - 38]		
≤30	119	(56.67)	60	(57.14)	59	(56.19)	0.889	18	(52.94)	0.668
>30	91	(43.33)	45	(42.86)	46	(43.81)		16	(47.06)	
**Marital status**										
Married/cohabiting	82	(39.05)	34	(32.38)	48	(45.71)	0.131	21	(61.76)	**0.009**
Divorced/separated/widowed	15	(7.14)	9	(8.57)	6	(5.71)		1	(2.94)	
Single	113	(53.81)	62	(59.05)	51	(48.57)		12	(35.29)	
**Education level**										
College or higher	67	(31.9)	31	(29.52)	36	(34.29)	0.716	17	(50)	0.092
High School	85	(40.48)	45	(42.86)	40	(38.1)		10	(29.41)	
Up to some/Incomplete High School	58	(27.62)	29	(27.62)	29	(27.62)		7	(20.59)	
**Income (Colombian minimum wage 2010-2014)**										
More than 4 wage	9	(4.29)	2	(1.9)	7	(6.67)	0.083	5	(14.71)	0.004
More than 1 and up to 4 wage	79	(37.62)	35	(33.33)	44	(41.9)		15	(44.12)	
Up to 1 wage	120	(57.14)	66	(62.86)	54	(51.43)		14	(41.18)	
Do not know/ no answer	2	(0.95)	2	(1.9)	0	(0)		0	(0)	
**Social Stratum**										
Stratum 1 or 2	142	(67.62)	74	(70.48)	68	(64.76)	0.600	19	(55.88)	0.290
Stratum 3	53	(25.24)	25	(23.81)	28	(26.67)		12	(35.29)	
Stratum 4 or more	15	(7.14)	6	(5.71)	9	(8.57)		3	(8.82)	
**Age of first sexual intercourse (years)**										
Median [IQR]	17 [15 - 18]		17 [15 - 19]		16 [15 -18]			17 [16 -18]		
< 16	103		49		54		0.123	16		0.170
17 - 19	72		33		39			15		
> 20	35		23		12			3		
**Lifetime sexual partners**										
Median [IQR]	4 [2 - 6]		3 [2 - 6]		4 [3 - 6]			4 [3 - 7]		
1 - 2	56		32		24		0.456	8		0.718
3 - 4	64		30		34			10		
≥ 5	90		43		47			16		
**Parity**										
Median [IQR]			1 [0 - 2]		1 [0 - 2]			1 [0 - 2]		
Never	63		33				0.612	9		0.841
1 - 2	116		59					20		
≥ 3	31		13					5		
**Oral contraceptive intake (years)**										
Median [IQR]	3 [1 - 6]		3 [1 - 6]		4 [1 - 7]			4 [1 - 6]		
Never	45		21		24		0.608	8		0.781
< 5	81		44		37			12		
≥5	84		40		44			14		
**Frequency of cytology use**										
Once or more than once every year	129	(61.43)	67	(63.81)	62	(59.05)	0.660	15	(44.12)	0.146
Once every 2-3 years	51	(24.29)	24	(22.86)	27	(25.71)		12	(35.29)	
Once every 4-5 years	22	(10.48)	9	(8.57)	13	(12.38)		6	(17.65)	
Do not know/no answer	8	(3.81)	5	(4.76)	3	(2.86)		1	(2.94)	
**hrHPV types frequency**										
HPV 16 and/or 18	87	(41.43)	34	(32.38)	53	(50.48)	**0.008**	20	(58.82)	**0.006**
Other hrHPV types[c]	123	(58.57)	71	(67.62)	52	(49.52)		14	(41.18)	

Abbreviations: ≤CIN1 Low-grade neoplasia (NEG and CIN1); NEG: Negative or without lesion; CIN1: Cervical intraepithelial neoplasia grade 1; CIN2+: High-grade neoplasia (CIN2 and CIN3); CIN2: Cervical intraepithelial neoplasia grade 2; CIN3: Cervical intraepithelial neoplasia grade 3; IQR: Interquartile range.

Person's chi-squared test for [a]≤CIN1 vs CIN2, and [b]≤CIN1 vs CIN3+.

[c]Other hrHPV types = HPV 31, 33, 35, 39, 45, 51, 52, 56, 58, 59, 66, 68.
