# Supplementary material for: miRNAs signature as potential biomarkers for cervical precancerous lesions in human papillomavirus positive women
Source: Sci Rep. 2023 Jun 17;13:9822. doi: 10.1038/s41598-023-36421-9 (PMC10276834; doi:10.1038/s41598-023-36421-9)
Supplement: Supplementary file 6 — Supplementary Table 3. [file 41598_2023_36421_MOESM6_ESM.pdf]

Supplementary Table 3. Fold change and Coefficient variation of the miRNAs differentially expressed between CIN2+ and ≤CIN1; or only CIN3 and ≤CIN1.

	**CIN2+ (n=10)** **(CIN2 n =7 and CIN3 n = 3)**		**Only CIN3 (n=3)**		
**miRNA**	**FC**	**p-value**	**FC**	**p-value**	**% CV**
**miR-133a-3p**	**6.9**	**<0.001**	**3.5**	**0.001**	**44**
**miR-143-5p**	**5**	**<0.001**	**2.7**	**0.008**	**30**
**miR-143-3p**	**5.1**	**<0.001**	**2.6**	**0.011**	**19**
miR-145-5p	4.8	<0.001	2.5	0.018	22
miR-145-3p	4.5	<0.001	2.3	0.032	30
miR-1-3p	4.4	<0.001	3.1	0.002	48
miR-9-3p	3.3	<0.001	1.9	0.079	26
miR-99a-3p	3.4	0.001	1.9	0.096	25
miR-376b-3p	3.8	0.001	2	0.069	36
miR-125b-2-3p	2.8	0.001	1.7	0.145	21
let-7c-5p	3	0.001	1.9	0.095	13
miR-4329	2.4	0.002	1.6	0.149	18
**miR-29a-3p**	**2.6**	**0.002**	**1.7**	**0.137**	**10**
miR-28-3p	2.5	0.002	1.9	0.066	13
miR-125b-5p	2.9	0.002	1.7	0.158	12
miR-199a-5p	3.1	0.002	1.6	0.214	18
miR-9-5p	3.2	0.002	1.7	0.15	25
miR-214-5p	3.1	0.002	1.6	0.205	29
miR-619-3p	2.1	0.002	1.4	0.281	15
miR-381-3p	3.2	0.002	1.7	0.144	28
miR-195-3p	2.9	0.002	1.6	0.197	27
**miR-30b-5p**	**2.1**	**0.002**	**1.7**	**0.085**	**9**
miR-99a-5p	2.8	0.003	1.6	0.184	14
miR-204-5p	2.9	0.003	1.9	0.089	25
miR-10b-3p	2.4	0.003	1.8	0.111	19
miR-133b	3	0.003	2.3	0.022	24
miR-1271-5p	2.3	0.003	1.5	0.247	18
miR-100-5p	2.7	0.003	1.7	0.158	16
miR-139-5p	2.6	0.003	1.8	0.108	23
miR-130a-3p	2.3	0.004	1.5	0.214	12
miR-196b-5p	2.8	0.004	1.8	0.111	18
miR-376a-3p	3.2	0.004	1.7	0.151	32
miR-134-5p	2.7	0.004	2	0.073	25
miR-497-3p	3	0.004	1.6	0.221	32
miR-10b-5p	2.7	0.004	1.7	0.17	16
miR-323a-3p	2.5	0.004	1.6	0.202	22
miR-195-5p	2.9	0.005	1.6	0.206	19
miR-379-5p	2.9	0.005	1.9	0.098	26
miR-7976	2.1	0.005	1.5	0.172	15
miR-542-5p	2.3	0.005	1.8	0.094	19
miR-4324	2.4	0.005	1.5	0.295	21
miR-99b-3p	1.9	0.005	1.4	0.262	14
miR-382-5p	2.7	0.006	1.8	0.109	26
miR-199b-5p	2.8	0.006	1.5	0.254	17

miR-494-3p	2.5	0.006	1.9	0.079	23
miR-140-5p	2	0.006	1.8	0.078	12
let-7f-2-3p	2	0.006	1.3	0.435	15
miR-126-3p	2.1	0.006	1.5	0.219	8
miR-199a-3p	2.7	0.006	1.6	0.245	14
miR-27a-3p	2.2	0.006	1.6	0.19	11
miR-154-5p	2.8	0.006	1.6	0.203	30
miR-654-3p	2.7	0.006	1.8	0.128	26
miR-337-5p	2.7	0.006	1.8	0.136	27
miR-409-5p	2.2	0.007	1.7	0.118	20
miR-342-5p	1.9	0.007	1.7	0.078	15
miR-214-3p	2.4	0.007	1.4	0.359	20
miR-299-5p	2.4	0.007	1.5	0.308	23
miR-196a-5p	2.4	0.007	2.1	0.043	21
let-7i-3p	1.7	0.007	1.3	0.41	11
miR-1296-5p	2.3	0.008	1.6	0.205	21
miR-27b-3p	2.4	0.008	1.6	0.187	12
miR-3120-3p	2.1	0.008	1.5	0.216	18
miR-126-5p	2	0.008	1.4	0.348	9
miR-26a-2-3p	1.9	0.008	1.4	0.301	14
miR-505-3p	1.5	0.008	1.4	0.173	8
miR-29c-3p	2	0.009	1.4	0.278	8
miR-136-5p	2.8	0.009	1.6	0.221	30
miR-199b-3p	2.7	0.009	1.5	0.262	18
miR-136-3p	2.6	0.01	1.5	0.251	26
miR-411-5p	2.7	0.01	1.7	0.163	29
miR-497-5p	2.7	0.01	1.5	0.262	24
miR-499a-5p	2	0.011	1.2	0.643	17
miR-365a-3p	2.1	0.011	1.5	0.264	17
miR-299-3p	2.4	0.011	1.7	0.162	24
miR-3928-5p	1.7	0.011	1.5	0.194	13
miR-28-5p	2.1	0.012	1.5	0.252	18
miR-26a-5p	1.8	0.012	1.3	0.387	6
miR-152-3p	2	0.012	1.5	0.212	12
miR-504-5p	2.3	0.012	1.8	0.121	24
miR-187-3p	2.4	0.012	2	0.067	21
miR-30e-3p	1.7	0.012	1.5	0.143	8
miR-125a-5p	2.1	0.013	1.5	0.254	10
miR-127-5p	2.4	0.013	2	0.065	26
miR-376c-3p	2.7	0.013	1.5	0.307	29
miR-29b-3p	1.8	0.014	1.3	0.438	8
miR-6885-5p	2.1	0.014	1.8	0.098	19
miR-542-3p	2.5	0.014	1.7	0.161	26
let-7e-5p	2	0.014	1.5	0.227	11
miR-146b-5p	1.8	0.014	1.1	0.884	9
miR-125b-1-3p	2	0.014	1.5	0.268	18
miR-342-3p	1.7	0.015	1.3	0.385	8
miR-151b/151a-	1.8	0.015	1.5	0.175	11
miR-409-3p	2.4	0.015	1.7	0.144	26

miR-1268a	1.7	0.015	1.3	0.333	14
miR-212-3p	1.8	0.015	1.5	0.176	14
miR-574-3p	2	0.015	1.5	0.239	13
miR-335-5p	1.9	0.015	1.4	0.296	11
miR-151a-3p	1.7	0.016	1.6	0.115	8
miR-374b-5p	1.5	0.017	1.3	0.303	7
miR-365b-3p	2	0.017	1.3	0.446	17
miR-590-5p	1.6	0.017	1.3	0.273	11
miR-127-3p	2.5	0.017	1.7	0.159	24
miR-889-3p	1.9	0.018	1.7	0.118	17
miR-4532	2.4	0.018	2	0.062	27
miR-1292-5p	2	0.018	1.5	0.242	14
miR-23a-3p	1.8	0.018	1.3	0.344	8
miR-99b-5p	1.9	0.018	1.6	0.163	10
miR-3175	1.6	0.019	1.5	0.135	13
miR-3934-5p	1.9	0.019	1.8	0.069	16
miR-487b-3p	2.4	0.019	1.7	0.169	30
miR-377-3p	2.5	0.019	1.3	0.468	33
miR-30c-5p	1.5	0.019	1.4	0.146	6
miR-376c-5p	2.4	0.02	1.6	0.231	31
miR-26b-5p	1.5	0.02	1.3	0.236	4
miR-369-5p	2.4	0.02	1.6	0.22	31
miR-663a	2.2	0.021	1.6	0.235	23
miR-24-3p	1.9	0.021	1.4	0.284	9
miR-23b-3p	2	0.022	1.4	0.332	11
miR-338-3p	1.8	0.022	1.6	0.141	13
miR-361-5p	1.7	0.022	1.3	0.446	9
miR-2117	1.6	0.023	1.3	0.362	12
miR-132-5p	1.7	0.024	1.3	0.389	14
miR-26b-3p	1.5	0.025	1.3	0.284	10
miR-339-5p	1.4	0.026	1.5	0.104	7
miR-455-3p	2.2	0.026	1.4	0.39	24
miR-590-3p	1.6	0.027	1.2	0.604	10
miR-887-3p	1.6	0.028	1.7	0.053	12
miR-329-3p	1.7	0.03	1.1	0.87	14
let-7a-3p	1.7	0.03	1.4	0.334	13
miR-101-3p	1.6	0.03	1.4	0.266	6
miR-27a-5p	1.5	0.03	1.3	0.363	10
miR-361-3p	1.5	0.032	1.4	0.266	8
miR-769-5p	1.6	0.033	1.7	0.052	11
miR-30d-5p	1.4	0.033	1.5	0.075	5
miR-2277-3p	1.8	0.033	1.3	0.4	11
miR-186-5p	1.4	0.033	1.3	0.203	5
miR-3656	1.7	0.035	1.7	0.089	15
let-7g-3p	1.8	0.035	1.5	0.226	13
miR-503-5p	1.9	0.036	1.7	0.143	17
miR-26a-1-3p	1.6	0.036	0.6	0.163	19
miR-4721	1.6	0.037	1.3	0.394	12
miR-184	1.8	0.037	1.7	0.117	18

miR-670-5p	1.7	0.039	1.6	0.171	22
miR-598-3p	1.3	0.039	1.4	0.118	6
miR-4322	2	0.039	1.9	0.09	23
miR-140-3p	1.5	0.041	1.7	0.046	6
miR-874-3p	1.6	0.041	1.4	0.288	13
miR-370-3p	1.6	0.041	1.6	0.114	14
miR-622	1.6	0.041	1.5	0.194	13
miR-455-5p	2.1	0.042	1.3	0.538	20
miR-3666	1.9	0.043	1.5	0.236	20
miR-33a-5p	1.7	0.043	1.1	0.736	15
miR-574-5p	1.6	0.044	1.5	0.175	13
miR-6808-3p	1.6	0.045	1.3	0.421	11
miR-1248	1.4	0.046	1	0.974	14
miR-34a-5p	1.7	0.046	1.3	0.408	9
miR-374a-3p	1.6	0.048	1.2	0.469	12
miR-675-3p	1.7	0.049	1.5	0.247	16
miR-6817-3p	1.6	0.049	1.1	0.779	13
miR-103a-2-5p	1.6	0.05	1.6	0.15	15
miR-432-5p	1.7	0.05	1.4	0.367	14
miR-29a-5p	1.5	0.05	1.3	0.407	11

FC: Fold Change between CIN2+ and ≤CIN1; and CIN3+ and ≤CIN1.

p value Walt test - DESeq2 algorithm.
