# Supplementary material for: miRNAs signature as potential biomarkers for cervical precancerous lesions in human papillomavirus positive women
Source: Sci Rep. 2023 Jun 17;13:9822. doi: 10.1038/s41598-023-36421-9 (PMC10276834; doi:10.1038/s41598-023-36421-9)
Supplement: Supplementary file 7 — Supplementary Table 4. [file 41598_2023_36421_MOESM7_ESM.pdf]

Supplementary Table 4. Fold changes and coefficient variations of the difference between the average dCt values of CIN2+ or CIN3+ and the average dCt values of the reference group ($\leq$CIN1) for each miRNA validated by RT-qPCR.

	CIN2+		CIN3+		
**miRNA**	**FC**	**p-value**	**FC**	**p-value**	**% CV**
miR-29a-3p	0,9	0,893	1,1	0,286	4,9
miR-30b-5p	1,3	0,123	1,7	0,077	14,5
miR-133a-3p	0,9	0,842	1,5	0,329	0,4
miR-143a-3p	1,2	0,093	1,8	0,057	5,9
**miR-143a-5p**	1,5	**0,021**	1,8	0,144	0,6

FC = $2^{-\Delta\Delta Ct}$. Where ΔΔCt is the difference between average ΔCt in CIN2+ or CIN3+ and the average of ΔCt values of the reference group (≤CIN1), for each miRNA. p-value U Mann Whitney test. $p<0.05$ in bold. CV: Coefficient of variation (n = 210 samples) of the normalized Ct values of each miRNA.
