# Supplementary material for: miRNAs signature as potential biomarkers for cervical precancerous lesions in human papillomavirus positive women
Source: Sci Rep. 2023 Jun 17;13:9822. doi: 10.1038/s41598-023-36421-9 (PMC10276834; doi:10.1038/s41598-023-36421-9)
Supplement: Supplementary file 8 — Supplementary Table 5. [file 41598_2023_36421_MOESM8_ESM.pdf]

Supplementary Table 5. Number and percentages of ≤CIN1 controls and CIN2+ and CIN3+ cases with negative or positive tests results**.**

	**≤CIN1 (n=79)**		**CIN2+ (n=79)**		**CIN3+ (n=27)**	
	TN n (%)	FP n (%)	TP n (%)	FN n (%)	TP n (%)	FN n (%)
5 miRNA signature ≥0.54	56 (70.88)	23 (29.11)	41 (51.90)	38 (48.10)	14 (51.85)	13 (48.15)
HPV16/18[a]	47 (65.28)	25 (34.72)	40 (51.28)	38 (48.72)	16 (59.26)	11 (40.74)
5 miRNA signature ≥0.54 plus HPV16/18	38 (48.10)	41 (51.90)	61 (77.22)	18 (22.78)	23 (85.19)	4 (14.81)

Positive test cut-off for the 5 miRNAs signature ≥0.54 normalized relative Ct values [a]Positive for any of the two tests. 2 CIN3+ and 1 CIN2 cases and 7 ≤CIN1 controls have missed results for HPV16/18. TP=true positive. FN=false negative. TN=true negative. FP=false positive.
