# Supplementary material for: miRNAs signature as potential biomarkers for cervical precancerous lesions in human papillomavirus positive women
Source: Sci Rep. 2023 Jun 17;13:9822. doi: 10.1038/s41598-023-36421-9 (PMC10276834; doi:10.1038/s41598-023-36421-9)
Supplement: Supplementary file 9 — Supplementary Table 6. [file 41598_2023_36421_MOESM9_ESM.pdf]

Supplementary Table 6. Diagnostic performance of the 5 miRNA signature or HPV 16/18 genotyping or their combination for detection of CIN2+ and CIN3+

	≤CIN1 (n=79)		CIN2+ (n=79)		CIN3+ (n=27)	
	Specificity, % (95%CI)	p value*	Sensitivity, % (95%CI)	p value*	Sensitivity, % (95%CI)	p value*
5 miRNA signature ≥0,54	70.88 (59.58–80.57)	NA	51.90 (40,36-63,30)	NA	51.85 (31.95–71.33)	NA
HPV16/18[a]	65.28 (53.14–76.12)	0,7194	51.28 (39.69–62.77)	0,8759	59.26 (38.80–77.61)	0,8026
5 miRNA signature ≥0,54 plus HPV16/18	49.73 (42.48–56.97)	**0,0001**	77.22 (66.40-85.90)	**<0.0001**	85.19 (66.27–95.81)	**0,0076**

Positive test cut-off for the 5 miRNAs signature at ≥0,54 normalized relative Ct values. [a]Positive for any of the two tests. 2 CIN3+ and 1 CIN2 cases and 7 ≤CIN1 controls have missed results for HPV16/18. Only paired results were included in the McNemar test. *p value of McNemar test for comparison of the 5 miRNA signature at ≥0.54 with each other tests. $p<0.05$ in bold. NA: Not Applicable: test same comparison.
