# Supplementary material for: miRNAs signature as potential biomarkers for cervical precancerous lesions in human papillomavirus positive women
Source: Sci Rep. 2023 Jun 17;13:9822. doi: 10.1038/s41598-023-36421-9 (PMC10276834; doi:10.1038/s41598-023-36421-9)
Supplement: Supplementary file 10 — Supplementary Table 7. [file 41598_2023_36421_MOESM10_ESM.pdf]

Supplementary Table 7. Target gene products with observed validated inverse correlations with expression of corresponding miRNA**.**

miRNA	FC (CIN2+/≤CIN1)	p-value	No. of target genes	Target gene products
miR-133a-3p	6.9	<0.001	15	CCN2, CDK13, FSCN1, IGF1R, KCNH2, KRT7, MCL1, NFATC4, PKM, PTPRK, RB1CC1, RHOA, RUNX2, SRF, STK3
miR-143-3p	5.1	<0.001	10	BCL2, DNMT3A, IGFBP5, **KRAS**, MAPK12, MAPK7, **MDM2**, PLK1, PRC1, TOP2A
miR-145-5p	4.8	<0.001	39	AHNAK, CCNA2, **CDK4,** CES1, CLINT1, DDR1, DFFA, EIF4E, EIF4EBP2, F11R, FSCN1, IGF1R, IRS1, KLF4, KLF5, KRT7, LAMP2, MAPK7, **MDM2**, MITF, MMP1, MUC1, MYC, MYO5A, NDUFA4, PADI1, PARP8, PPP3CA, RAB27A, RASA1, RTKN, SOX9, SPTB, SWAP70, TPM3, TYR, TYRP1, UNG, USP46
miR-1-3p	4.4	<0.001	103	ADAR, ADPGK, AGRN, ANPEP, ANXA2, AP3B1, AP3D1, ARCN1, ARF3, ARF4, ARHGEF18, ARID1A, ARID2, ASH2L, ATP6V0A1, ATP6V1B2, AXL, BCKDHB, BCL2, BDNF, CAND1, CAP1, CDK14, CDK9, CHST11, CHSY1, CLCN3, CNOT6, CPOX, CTSC, DDX5, DHX15, DNAJB1, **EGFR**, EHMT1, ESR1, F2, FERMT2, G6PD, GAK, GCH1, GJA1, GNPDA2, GNPNAT1, GPD2, H3-3A/H3-3B, HACD2, HACD3, HAND2, HDAC4, IGF1, INPP5F, IP6K2, IQGAP3, ISY1, **ITGB4**, KCNJ2, LASP1, LRP1, LRRC8A, MEF2A, MET, MRC2, MTHFD2, **NOTCH2, NOTCH3**, NRP1, OAT, PDCD4, PDLIM7, PGM2, PICALM, PICALM, PNP, POLA1, POLA2, POLR2K, POM121/POM121C, PPIB, PREX1, PRSS21, PTBP1, PTPRF, PXYLP1, RAB11FIP2, SDC4, SERPINB5, SPHK1, TAC1, TDP1, **THBS1**, TIMP3, TMSB10/TMSB4X, TPM1, TPM2, TPM3, TPM4, TSPAN4, TWF1, UHMK1, UHRF1, UST, UTRN, YWHAQ
miR-9-3p	3.3	<0.001	1	RCOR1
let-7c-5p	3	0.001	97	AARSD1, ACP1, ADAMTS14, ADAMTS15, ADAMTS2, ADGRG1, AGO4, AKAP8, ANAPC1, ATP6V0A1, ATP6V1F, AURKB, BCL2L1, BSG, **CASP3, CCND1**, CDC25A, CDIPT, **CDK6**, CEMIP2, CHMP2A, CIAO2A, COL1A1, COL1A2, COL4A1, COL4A2, COL4A5, COL5A1, COL5A2, COL8A1, CSDE1, CSNK1D, DICER1, DRD3, DSP, DUSP12, DUSP23, EIF3J, EIF4G2, F2, FADS2, FANCD2, GAK, GYS1, HMGA1, HMGA2, HMOX1, IGF2BP1, **ITGB3**, KCNJ16, KLK10, **KRAS**, KRT19, **LAMC1**, LIN28A, MARS2, MYC, NEDD4, NF2, **NRAS,** NXN, PDGFA, PDGFB, POLD2, POLR2C, POM121/POM121C, PPP1R7, PRDM1, PRIM1, **PTGS2**, RAS, RDH10, RHOB, RHOB, RHOG, SEPTIN3, SIGMAR1, SLC1A4, SLC25A13, SLC38A1, SMC1A, SMOX, SNAP23, SPCS3, TAF9B, TAGLN, TGFBR1, TGFBR2, **THBS1**, TLR4, TPM2, TYMS, UGT8, UHRF1, VCAN, VIM, VPS39, **WNT1**
miR-29a-3p	2.6	0.002	52	ACVR2A, ADAMTS14, ADAMTS15, ADAMTS2, ARPC3, BACE1, CAV2, **CDC42**, **CDK6**, CNOT8, COL15A1, COL1A1, COL1A2, COL3A1, COL4A1, COL4A2, COL4A5, COL5A1, COL5A2, COL5A3, COL8A1, DCP2, DNMT3A, DNMT3B, DUSP2, EOMES, GPR37, HDAC4, INSIG1, KLF4, **LAMC1,** MAPRE2, MCL1, MYBL2, PDGFA, PDGFB, PIK3R1, PPM1D, **PTEN**, SP1, TBX21, TCL1A, TDG, TET1, TGFB3, TGFBR1, TGFBR2, TNFAIP3, TUBB2A, VCAN, YY1, ZFP36L1
miR-28-3p	2.5	0.002	1	AR
miR-125b-5p	2.9	0.002	51	ACSS1, ADAMTS1, AJUBA, ALOX5, APLN, BAK1, BMPR1B, CASP6, CASP7, CBX7, CCR5, CDC25A, CDH5, **CDK6**, CDKN2A, CEBPG, CYP1A1, DDX19B, DICER1, DIO3, E2F3, ELAVL1, ENTPD4, ERBB2, ERBB3, GPR160, GSS, H3-3A/H3-3B, HK2, ID1, ID2, ID3, IGFBP3, IL1RN, JARID2, KRT19, LIN28A, LIPA, MAP2K7, MYD88, PERP, RBM8A, SCD, SH3BP4, SMO, **TP53**, UBE2I,
miR-199a-5p	3.1	0.002	9	ALOX5AP, DYRK1A, ETS1, HIF1A, **LAMC2**, MYH9, PDCD4, SET, SIRT1
miR-9-5p	3.2	0.002	14	BACE1, CDH1, FGF16, FOXG1, **FOXO1**, HSP90AA1, **JAK1**, JAK2, JAK3, **NFKB1**, NTRK3, PRDM1, REST, ZFP36
miR-30b-5p	2.1	0.002	72	ACVR1, ADAMTS14, ADAMTS15, ADAMTS2, ADPGK, ANPEP, AP2A1, ATP2A2, ATRX, BCL6, BECN1, CCN2, CNOT9, COL1A1, COL1A2, COL4A1, COL4A2, COL4A5, COL5A1, COL5A2, COL8A1, F2, GNAI2, GPD2, IDH1, **ITGA2**, JUN, KRT7, KRT85, **LAMC1**, LMNB2, LRRC8C, MAP4K4, MAT2A, MET, MLLT11, MYO10, NAPG, NCL, NEUROD1, NPR3, NT5C3A, NT5E, PAFAH1B2, PDGFA, PDGFB, PGM1, PNP, PPP3CA, PPP3R1, PRPF40A, PTPA, PTPRK, RAD23B, RUNX2, SLC12A4, SLC38A1, SLC4A10, SLC7A1, SLC7A11, STX1A, SYT4, **TCF7**, TGFBR1, TGFBR2, THEM4, TNFRSF10B, **TP53**, UAP1, UBE2I, VCAN, WNT5A, WNT5A
miR-99a-5p	2.8	0.003	7	FGF16, FGFR3, IGF1R, **MTOR**, PLK1, RPTOR, SMARCA5
miR-204-5p	2.9	0.003	16	ARPC1B, ATP2B1, AURKB, BMP1, CDC25B, CDH11, CTSC, EFNB1, HMGA2, **ITGB4**, MMP3, MMP9, SHC1, SOX4, SPDEF,

Ingenuity Pathway Analysis (QIAGEN Inc., https://digitalinsights.qiagen.com/IPA) showing target gene products with validated inverse correlation for 14 of the top 25 differentially expressed miRNAs. In bold the related genes in the list of Human Papillomavirus Infection from KEGG (26/231 genes) (https://www.genome.jp/pathway/ko051659).
